# Phrenic nerve deficits and neurological immunopathology associated with acute West Nile virus infection in mice and hamsters

**DOI:** 10.1007/s13365-016-0488-6

**Published:** 2016-10-19

**Authors:** Katherine Zukor, Hong Wang, Brett L. Hurst, Venkatraman Siddharthan, Arnaud Van Wettere, Paul M. Pilowsky, John D. Morrey

**Affiliations:** 1 Institute for Antiviral Research, Department of Animal, Dairy, and Veterinary Sciences, 5600 Old Main Hill, Utah State University, Logan, UT 84322-5600 USA; 2 Utah Veterinary Diagnostics Laboratory, Department of Animal, Dairy, and Veterinary Sciences, 5600 Old Main Hill, Utah State University, Logan, UT 84322-5600 USA; 3 The Heart Research Institute and Department of Physiology, University of Sydney, Sydney, NSW Australia

**Keywords:** West Nile virus, Breathing, Respiratory, Phrenic, Electrophysiology, Sequelae

## Abstract

Neurological respiratory deficits are serious outcomes of West Nile virus (WNV) disease. WNV patients requiring intubation have a poor prognosis. We previously reported that WNV-infected rodents also appear to have respiratory deficits when assessed by whole-body plethysmography and diaphragmatic electromyography. The purpose of this study was to determine if the nature of the respiratory deficits in WNV-infected rodents is neurological and if deficits are due to a disorder of brainstem respiratory centers, cervical spinal cord (CSC) phrenic motor neuron (PMN) circuitry, or both. We recorded phrenic nerve (PN) activity and found that in WNV-infected mice, PN amplitude is reduced, corroborating a neurological basis for respiratory deficits. These results were associated with a reduction in CSC motor neuron number. We found no dramatic deficits, however, in brainstem-mediated breathing rhythm generation or responses to hypercapnia. PN frequency and pattern parameters were normal, and all PN parameters changed appropriately upon a CO_2_ challenge. Histological analysis revealed generalized microglia activation, astrocyte reactivity, T cell and neutrophil infiltration, and mild histopathologic lesions in both the brainstem and CSC, but none of these were tightly correlated with PN function. Similar results in PN activity, brainstem function, motor neuron number, and histopathology were seen in WNV-infected hamsters, except that histopathologic lesions were more severe. Taken together, the results suggest that respiratory deficits in acute WNV infection are primarily due to a lower motor neuron disorder affecting PMNs and the PN rather than a brainstem disorder. Future efforts should focus on markers of neuronal dysfunction, axonal degeneration, and myelination.

## Introduction

About 20 % of people infected with West Nile virus (WNV) develop fever with headache, diarrhea, body and joint aches, or rash. Less than 1 % of patients who become infected develop a serious neurological disease during the acute phase that is characterized by neck stiffness, disorientation, respiratory distress, tremors, paralysis, and seizures. Some of these neurological sequelae may persist for long periods or become permanent (Carson et al. 2006; Sejvar et al. 2008; Sejvar et al. 2003). Here, we investigate the development of acute neurological respiratory deficits in WNV-infected rodents and provide insights into the physiological mechanisms of these deficits.

Neurological respiratory deficits are serious outcomes of WNV disease. Patients that require intubation have a poor prognosis (Sejvar et al. 2006). Studies in animal models suggest that respiratory failure may be a cause of death (Wang et al. 2013). Neurological respiratory deficits in WNV patients are associated with histopathologic lesions in motor neurons of the anterior spinal cord (Fratkin et al. 2004; Leis and Stokic 2012) as well the medullary brainstem (Sampson et al. 2000; Sampson and Armbrustmacher 2001), which contains several clinically relevant regions for breathing function. These include the retrotrapazoid nucleus (RTN) which responds to changes in CO_2_ (Dubreuil et al. 2009), the Bötzinger complex (BötC) which regulates breathing (Guyenet 2000; Pilowsky et al. 1990b), the pre-Bötzinger complex (preBötC) which contains the breathing rhythm generator (Smith et al. 1991; Sun et al. 2001; Sun et al. 1998), and the ventral respiratory group (VRG) which contains pre-motor neurons (Feldman et al. 1985; Sun et al. 1994) which transmit signals to the phrenic motor neurons (PMNs) (Pilowsky et al. 1990a) in the cervical spinal cord (CSC), which then innervate the diaphragm.

WNV-infected mice and hamsters are valuable models for the human WNV disease (Hunsperger and Roehrig 2006; Morrey et al. 2013; Xiao et al. 2001). Histopathologic lesions involving the brainstem and spinal cord are well documented in both mice (Hunsperger and Roehrig 2006; Szretter et al. 2009) and hamsters (Morrey et al. 2012; Wang et al. 2009a). Respiratory deficits in infected hamsters were first confirmed with electromyography (EMG) of the diaphragm (Morrey et al. 2010). A follow-up study (Morrey et al. 2012) suggested that the mechanistic cause of death in WNV-infected hamsters and mice may be respiratory insufficiency.

In another study in mice (Wang et al. 2013), the function of PMNs in the CSC was directly evaluated using optogenetics. EMG recordings after photostimulation of channel rhodopsin-expressing PMNs were diminished in WNV-infected mice compared to shams, suggesting that PMN function is impaired by WNV infection. Analysis of EMG activity during endogenous breathing suggested that brainstem respiratory functions may also be impaired. Diaphragmatic EMG amplitudes from WNV-infected mice were dramatically reduced or undetectable after a CO_2_ challenge. The caveat to this conclusion, however, is that baseline, room air EMG activity was absent for both sham and WNV groups; therefore, CO_2_ responses in terms of an increase from baseline could not be calculated. It is possible that WNV-infected animals can respond to CO_2_, but a more sensitive test is needed to detect it.

The goal of the current study was to determine if the respiratory abnormality following acute WNV infection in mouse and hamster is due to dysfunction of the CSC/PMNs, or of brainstem respiratory centers, or both. CSC/PMN function was assessed by recording activity directly from the phrenic nerve (PN) rather than from its target muscle. Brainstem respiratory function was assessed by analyzing responses to CO_2_, breathing rhythm/pattern parameters, and hypoglossal nerve (12N) activity. The hypoglossal nucleus receives inputs from brainstem respiratory centers and displays bursts of activity corresponding to inspiration that are independent of the activity of spinal cord circuitry. To assess morphological damage to neurological respiratory centers, tissue sections of the medulla and CSC were analyzed for histopathology (hematoxylin and eosin (H&E) stain), WNV immunoreactivity (IR), neuronal survival, and markers of inflammation (microglia, astrocytes, T cells).

## Materials and methods

### Animals and virus

**Fig. 1:**
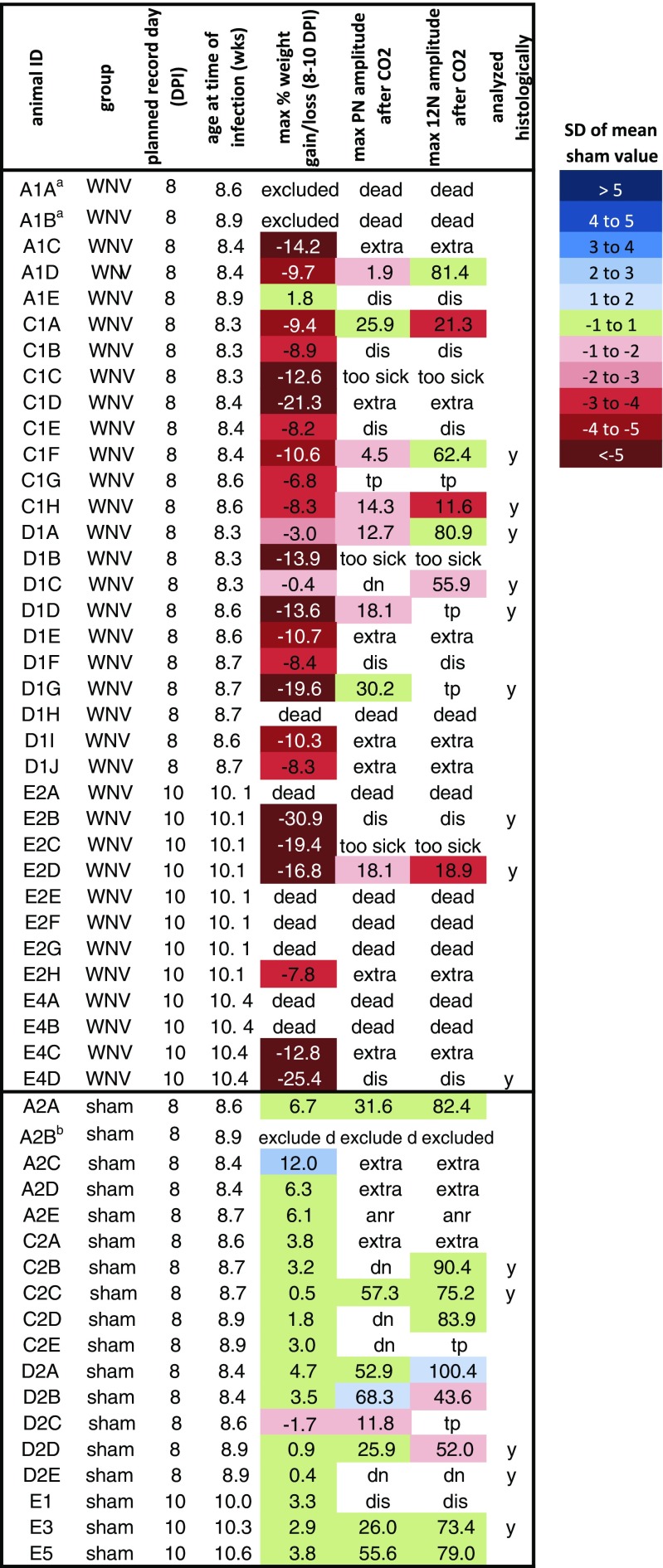
Overview of all animals entered into experiments, sorted by infection type (WNV or sham). First letter of mouse ID denotes experiment (A, C, D, or E). *Colors* categorize values in terms of standard deviations (SDs) of the sham mean. For example, if the value is over 1, but below 2 SD of the mean of the sham values for that parameter, the value is shown in the *lightest blue* (see “Materials and methods”). Animals indicated as “extra” were not needed because enough animals survived to the planned recording date. Animals that were very sick and not expected to survive the surgery were designated “too sick.” *tp* technical problem, *dis* died in surgery, *anr* apnea not reached, *dn* damaged nerve. *Superscript letter a*, excluded because underwent an unrelated trial procedure; *superscript letter b*, excluded because was a control with >10 % weight loss (color figure online)

This work was done in the Association for Assessment and Accreditation of Laboratory Animal Care International–accredited BSL3 laboratory at the Utah State University. All experimental procedures were performed in compliance with animal protocols approved by the Institutional Animal Care and Use Committee at Utah State University. Adult, wild-type C57BL/6 male mice (age > 7 weeks, Charles Rivers) were randomly assigned to treatment groups. WNV propagated in C6/36 mosquito cells and previously frozen was diluted to 4 pfu/μL with minimal essential medium supplemented with 50 μg/mL gentamicin (MEM + G) and injected subcutaneously into the footpad at a volume of 50 μL (200 pfu). The WNV isolate (strain WN02) recovered from a mosquito on 10 May 2007 in Kern County, CA, was provided by the University of Texas Medical Branch Arbovirus Reference Collection (TVP 10799 BBRC lot no. WNVKERN515-01). Sham-infected mice received a footpad injection of 50 μL of MEM + G. Four experiments, A, C, D, and E, were conducted to obtain nerve recording data. Figure 1 shows how many animals were in each group for each experiment, their ages at the time of infection, the planned day post-infection (DPI) for nerve recording, and which animals were included in the nerve recording and histological analyses. Infection dates were staggered so 1 mouse could undergo surgery each day on the intended DPI. Additional mice were infected with WNV to ensure at least one would be alive on the recording day. Even with this precaution, some preparations did not yield recordings because the most affected mice often did not survive the surgery. For the hamster study, LVG golden Syrian hamsters (female, 100 g) were obtained from Charles Rivers, randomly assigned to treatment groups, and infected with WNV (3.175 × 10^6^ pfu in 100 μL, inguinal subcutaneous injection, same WNV strain used in mice) or MEM.

### Nerve recording surgery

Nerve recording surgeries were performed as described previously (Farnham et al. 2015). Mice were anesthetized with urethane (1.3–1.5 g/kg i.p., 10 % solution in lactated Ringer solution (LRS), Sigma U2500). Urethane was used because it has minimal interference with nerve activity (Flecknell 2009). This full dose was split into two half-doses and given 15 min apart to ensure a smooth induction of anesthesia without hypotension. Atropine (0.015 mL i.p. of 400 μg/mL solution, West-ward, #NDC 0641-6006-01) was given with the first urethane injection to reduce bronchial secretions. Upon absence of a withdrawal reflex to paw pinch, animals were placed supine on a warming pad for surgery. Body temperature was monitored via a rectal probe (PhysioSuite, Kent Sci) and kept between 36.5 and 37.5 °C. Heart rate (HR) was monitored with a two-lead electrocardiogram (ECG) placed into the forepaws, with the ground wire being placed in the chest muscle. A tracheotomy was performed, and mice were ventilated (UgoBasile, #50094) with room air enriched with 100 % O_2_ at 1 L/min to saturate peripheral chemoreceptors in the carotid body (Hanna et al. 1981; Lahiri and DeLaney 1975). Tidal volume was 0.25–0.3 mL, and frequency was 250–270 bpm. These values are in the normal ranges for C57BL/6 mice (Berndt et al. 2012) and were usually sufficient to keep the mice in apnea until the beginning of nerve recording. End tidal CO_2_ was monitored (CapnoScan, Kent Scientific) with a sampling needle that was inserted slightly into the expired air-side of the tracheal tube. A cannula was then placed into the right external jugular vein, and LRS was given i.v. at a rate of about 0.5 mL/h. A neuromuscular blocking agent, rocuronium bromide (10 mL/mL, Mylan, #NDC 67457–228-05), and urethane were diluted 1/10 and 1/5 in LRS, respectively, and 50 μL of the combined solution was given every 25–30 min i.v. to achieve the equivalent of hourly 0.01- and 0.02-mL booster doses of the undiluted solutions, respectively. The neuromuscular blocking agent was given to prevent animals from breathing against the ventilator. Next, the vagus nerve on the right was cut, and the common carotid artery on the right was cannulated in order to monitor arterial blood pressure (BP, PendoTECH sensor, EW-19406-32, connected to ADInstrument’s Bridge amp, #FE117). The vagus nerve on the left was then cut to completely remove feedback from lung stretch receptors to the brainstem respiratory system. The left PN and 12N were then isolated from the ventral side of the animal and placed on bipolar silver hook electrodes for recording (perfluoroalkoxy-coated silver wire, 0.01″ bare, A-M Systems, #787000). After removing excess fluid from around the nerve, the nerve/hook assembly was insulated from the rest of the animal with a vaseline/oil mixture (25 % Vaseline™, 75 % paraffin oil, warmed to 37 °C).

### Nerve data acquisition

Activity recorded from nerves was acquired with a differential amplifier (Dagan EX4-400) with the following settings: 10,000× total gain, DC, A-B configuration, 100–10,000-Hz filter. Data were sampled at 4 kHz with PowerLab and viewed with its accompanying software, LabChart (ADInstruments). At the start of recoding, no activity was observed from either nerve due to respiratory alkalosis secondary to hyperventilation. To find the apneic threshold, the ventilator rate was lowered slowly (about 10 bpm/3 min) until stable activity in both nerves appeared. After stable activity was achieved, the ventilator was lowered another 10 bpm and kept steady for at least 10 min to record baseline values close to the apneic threshold. Then, animals were challenged with room air supplemented with 7 % CO_2_ balanced with O_2_ for 15 min.

### Nerve data analysis

**Fig. 2:**
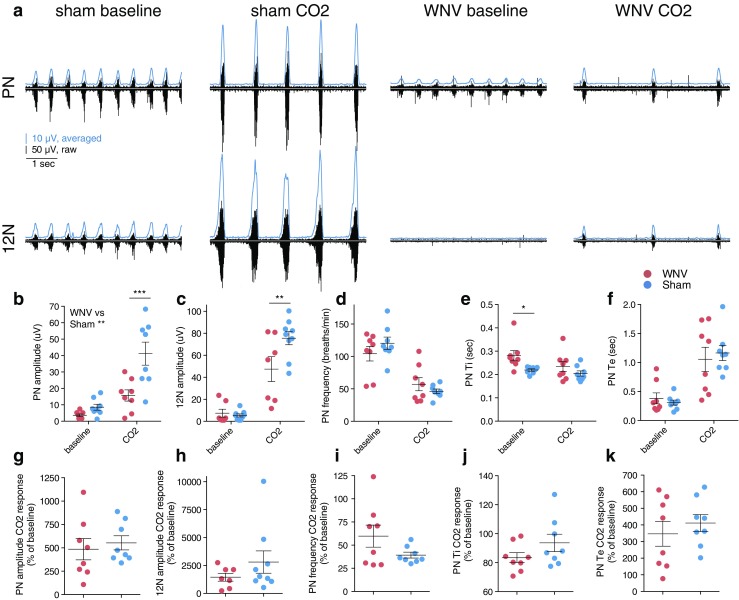
Nerve recordings at baseline and after CO_2_ challenge. **a** Representative PN and 12N recording traces from a WNV-infected (C1H) and sham-infected (C2C) mouse after 10 min at baseline and 15 min of 7 % CO_2_ challenge. Raw traces are *black* (50 μV scale), averaged traces are *blue* (10 μV scale). **b-f** Each trace represents 5 s. PN amplitude (**b**), 12N amplitude (**c**), and PN burst frequency (**d**), Ti (**e**), and Te (**f**). *Each dot* represents the average value for an animal. Separate values are shown for the 10-min baseline period and the end of the 15-min CO_2_ challenge period. Data were analyzed with two-way ANOVAs and post hoc *t* tests (*p ≤ 0.05; **p ≤ 0.01; ***p ≤ 0.005). **g**–**k** Values after the 15-min CO_2_ challenge period expressed as a percentage of baseline for each parameter: PN amplitude (**g**), 12N amplitude (**h**), and PN burst frequency (**i**), Ti (**j**), and Te (**k**). Data were analyzed with *t* tests. *Error bars* in **b**–**k** represent group mean ± SEM (color figure online)

**Fig. 3:**
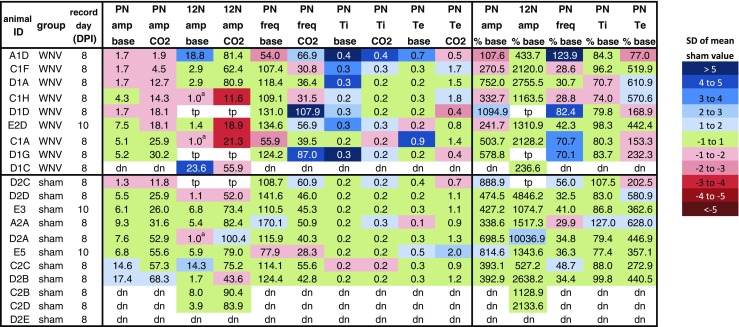
Heat map of nerve recording data for individual animals, sorted by infection type (WNV or sham) and PN amplitude after CO_2_ challenge. *Colors* categorize values in terms of standard deviations (SDs) of the sham mean. For example, if the value is over 1, but below 2 SD of the mean of the sham values for that parameter, the value is shown in the *lightest blue* (see “Materials and methods”). *base* baseline, *amp* amplitude, *freq* frequency, *tp* technical problem, *dn* damaged nerve. *Superscript letter a*, no 12N baseline (just noise) (color figure online)

Thirty seconds of data at the end of the 10 min baseline period and at the end of the 15 min CO_2_ challenge were analyzed for each animal using LabChart (ADI Systems; Figs. 2 and 3). Signals were digitally filtered (300–2000 Hz), rectified, and averaged (smoothing average, 0.1-ms time constant). Using the peak analysis tool, peaks in the averaged nerve signal were detected, and the average peak start time, end time, height (from baseline), and period were obtained. Peak start and end times were empirically found to correlate with raw burst start/end times at about 5 % of the height on either side of the peak. Time of inspiration (Ti) was calculated by subtracting peak start time from peak end time. Time of expiration (Te) was calculated by subtracting the next peak start time from the peak end time. Period was the peak-to-peak interval, and the burst frequency (bursts per minute) was calculated by dividing 60 by the period (in seconds). Two-way ANOVAs were used to analyze the main effects of WNV infection and CO_2_ level. Values after CO_2_ challenge were divided by baseline values to express post-challenge parameters in terms of percent of baseline. These data were compared with *t* tests. Mice were excluded from analysis if there was known damage to the nerve during isolation.

### Perfusion and tissue processing

After nerve recording was complete, mice were perfused transcardially with phosphate-buffered saline (PBS) followed by 4 % paraformaldehyde. The head and spinal column were removed and post-fixed in the same fixative overnight, rocking at 4 °C. Tissues were rinsed twice in PBS and dissected further. The spinal column was separated from the head by cutting between the C1/C2 vertebrae with a scalpel. After the brain was removed from the skull, it was put into a mouse brain slicer (ASI Instruments, RBM-2000C) and the medulla was isolated by cutting at the pons/medulla junction. To isolate spinal cord containing the PMNs, C2-C6 vertebrae were removed one-by-one, and the spinal cord was cut at the rostral border of the ventral C7 vertebral body and a small notch was made to identify the caudal end. The isolated piece of spinal cord corresponds to the C2-C7 spinal levels (Harrison et al. 2013). PMNs are located in C3-C5 (Watson et al. 2009). After brief storage in PBS at 4 °C, tissues were processed for paraffin embedding using Formula 83 (CBG Biotech, CH0104A) as a xylene substitute. The medulla and C2-C7 CSC were embedded in paraffin for cross sections, such that the first sections would be from the rostral side of the tissue.

### Immunohistochemistry and H&E staining

**Fig. 4:**
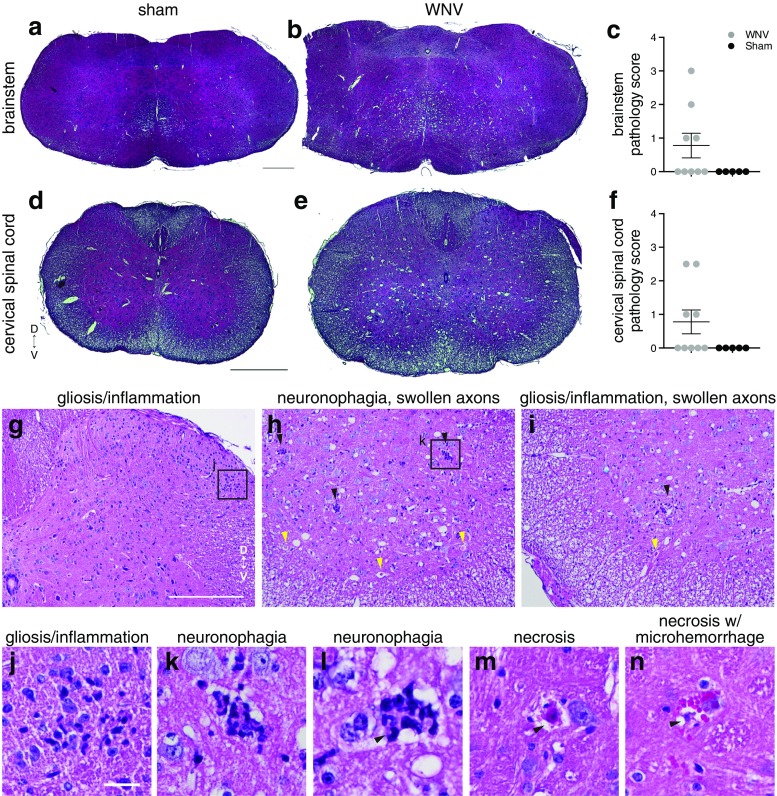
Histopathology, H&E staining. **a**, **b**, **d**, **e** Representative brainstem and CSC images from a sham-infected (E3) and a WNV-infected (E2B) mouse with histopathologic lesion scores of 3 in brainstem and 2.5 in CSC. Note the presence of vacuolated gray matter in WNV-infected sections. **c**, **f** Brainstem and CSC histopathologic lesion scores for individual animals in each group: 0 = no histopathology, 4 = severe. **g**–**n** Examples of lesions in WNV-infected animals. **g** Gliosis/inflammation in dorsal horn of spinal cord (in box). **h** Neuronophagia (*black arrowheads*) and swollen axons (*yellow arrowheads*) in ventral spinal cord. **i** Gliosis/inflammation (*black arrowhead*) and swollen axon (*yellow arrowhead*) in ventral spinal cord. **j** Close-up of gliosis/inflammation in box in **g**. **k** Close-up of neuronophagia in box in **h**. **l** Close-up of neuronophagia showing at least some of the cells surrounding the neuron being phagocytosed are neutrophils (*black arrowhead*). **m** Close-up of neuronal necrosis (*black arrowhead*). **n** Close-up of neuronal necrosis (*black arrowhead*) with microhemorrhage *(pink red blood cells*). *D* dorsal, *V* ventral. *Error bars* in **c** and **f** represent group mean ± SEM. *Scale bars* = 500 μm (**a**, **b** same scale, **d**, **e** same scale), 250 μm (**g**, **h**, **i** same scale), 25 μm (**j**–**n** same scale) (color figure online)

**Fig. 5:**
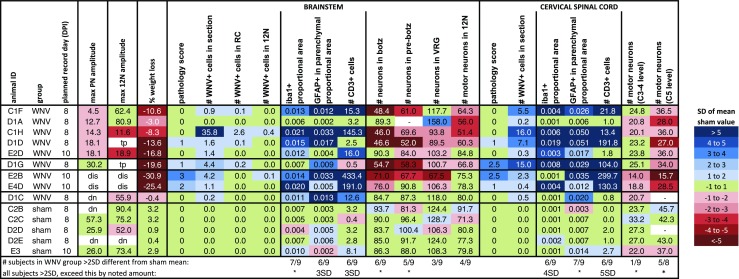
Heat map of immunohistologic results from each mouse, sorted by infection type (WNV or sham) and PN amplitude after CO_2_ challenge. *Colors* categorize values in terms of standard deviations (SDs) of the sham mean. For example, if the value is over 1, but below 2 SD of the mean of the sham values for that parameter, the value is shown in the *lightest blue* (see “Materials and methods”). *Asterisk* indicates WNV group mean significantly different from sham group mean (*p* < 0.05). *dis* died in surgery, *tp* technical problem, *dn* damaged nerve (color figure online)

Sections were cut on a microtome at a thickness of 5 μm. For brainstems, ribbons of sections were placed on black paper, and individual sections were floated on a 37 °C water bath in order to put adjacent sections onto 6 sets of slides. For CSCs, adjacent ribbons of three sections were mounted onto each of four to six sets of slides such that each ribbon on a slide represented 250 μm of tissue. The first set was stained with H&E (Figs. 4 and 5), and adjacent sets were used for immunohistofluorescent labeling with antibodies listed in Table 1. For all antibodies except for CD3, after dewaxing with xylenes and rehydration into water through a descending ethanol series, antigen retrieval was performed by incubating sections in Liberate Antigen Binding solution (Polysciences, Inc., 24310) for 30 min at room temperature (RT). After rinsing in water, slides were allowed to dry and sections were circled with a hydrophobic barrier pen (ImmEdge, Vector Labs). Sections were blocked with 10 % normal serum and 1 % Triton X-100 in PBS for 1 h. Primary antibodies were diluted in blocking solution as shown in Table 1 and applied to sections for incubation overnight at RT. Secondary antibodies conjugated to Alexa-488, Alexa-568, or Alexa-647 (from Invitrogen or Jackson ImmunoResearch) were diluted to 10 μg/mL in blocking solution. Sections were rinsed three times in PBS, incubated with secondary antibody solution for 2 h at RT, rinsed three times in PBS, incubated with Hoechst 33342 (Invitrogen, 1/2000 in PBS with 0.05 % Triton X-100), and rinsed twice in PBS. Coverslips were mounted with Fluoromount G (Southern Biotech). For CD3 labeling, antigen retrieval was performed in sodium citrate solution (Dako, S1699) in a pressure cooker (5 min at 1250 psi). After cooling to RT, sections were rinsed with water, encircled by a hydrophobic barrier, permeablized in PBS with 0.5 % Triton X-100 for 5 min, rinsed in PBS, and blocked in 10 % normal serum, 0.2 % Triton X-100 in PBS for 1 h. CD3 labeling was then identical to the method above except that primary and secondary antibodies were diluted in this blocking solution containing less Triton X-100.

**Table 1:** Primary antibodies

Antibody	Antibody type	Company, Catalog no.	Dilution
NeuN	Rabbit pAb	Millipore, ABN78	1/500
ChAT	Goat pAb	Millipore, AB144P	1/50
WNV	Human	Macrogenics, MGAWN1	1/500
5HT	Rabbit pAb	Immunostar, 20080	1/3000–1/6000
iba1	Goat pAb	Abcam, ab5076	1/200–1/600
GFAP	Rat IgG2a-kappa mAb	Invitrogen, 13-0300	1/500
CD3	Rabbit mAb	Abcam, ab16669	1/100

Summary of primary antibodies and dilutions used

*pAb* polyclonal antibody, *mAb* monoclonal antibody

### Imaging and image processing

Bright-field images were obtained with an Olympus BX 43 microscope. Fluorescent images were obtained with a laser scanning confocal microscope (Zeiss, LSM710) equipped with 405, 488, 561, and 633 laser lines and an automated stage. To obtain images of whole sections, multiple fields of view were scanned under 10X (Zeiss, Plan-APOCHROMAT) and stitched automatically. For images taken for pixel-based quantification, similar settings were used for all images in a set. Images were processed and analyzed with ImageJ or FIJI (Schneider et al., 2012) for quantification. For images chosen for publication, distracting artifacts were removed in ImageJ and levels were adjusted in Photoshop to maximize the signal-to-noise ratio so that relevant features could be seen more clearly. For images chosen to highlight pixel-based quantification, sham- and WNV-group images were adjusted identically to enable equitable comparison.

### Anatomical identification of brainstem regions

**Fig. 6:**
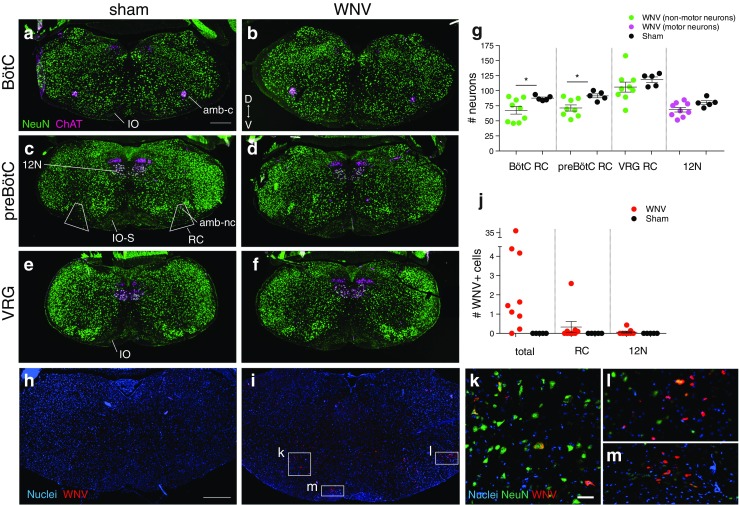
Neuronal survival and WNV IR in the brainstem. **a**–**f** Representative images of the brainstem at the level of the BötC, preBötC, and VRG from a WNV-infected (E4D) and sham-infected (E3) mouse labeled with antibodies against NeuN (*green*, all neurons) and ChAT (*magenta*, motor neurons). *IO* inferior olive, *amb-c* ambiguus nucleus compact, *12N* hypoglossal nucleus, *IO-S* S-shape of IO, *RC* respiratory column, *amb-nc* ambiguus nucleus noncompact. The wedges in **c** show which cells were included in RC counts in **g**. **g** Average number of neurons per section in each region. For RC regions, only nonmotor neurons were counted (*green*). For 12N, motor neurons were counted (*magenta*). Results were significantly different between groups for BötC RC and preBötC RC (*p < 0.05). **h**, **i** Representative images of the brainstem from a sham-infected animal and the WNV-infected animal having the most WNV IR of all mice (animal C1H). Sections were labeled with antibodies against WNV (*red*) and NeuN (*green*, shown in **k**–**m**). **k**–**m** Close-ups of WNV IR cells in **i** that include examples of infected cells with neuronal (**k**) and glial (**l**, **m**) morphology, and the location in the spinal trigeminal nucleus where WNV IR was frequently localized in animal C1H (**l**). **j** Average number of WNV IR cells per section for the whole section (total) and in each region (RC, 12N). *D* dorsal, *V* ventral. Scale bars = 500 μm (**a**–**f** same scale, **h**, **i** same scale), 50 μm (**l–m** same scale). *Error bars* in **g** and **j** represent group mean ± SEM. The *error bar* for total WNV+ cells is not shown because the *y*-axis is split into two segments and some of the data points are in segment 2 (color figure online)

Analysis of the medulla was confined to regions between the caudal border of the facial nucleus (7N) and the decussation of the pyramidal tracts. This region contains the RTN, BötC, PreBötC, and VRG. Sections on each slide set were assigned an anatomical level based on landmarks seen on the set of slides labeled with the neuronal marker, NeuN, and the motor neuron marker, choline acetyltransferase (ChAT). Key brainstem landmarks were the 7N, nucleus ambiguus compact (amb-c), nucleus ambiguus noncompact (amb-nc), 12N, and the shape of the inferior olivary nucleus (IO) (Fig. 6a, c, e). The BötC/RTN level included sections caudal to the 7N and rostral to the S-shape in the inferior olive (IO-S), generally where the amb-c is (Fig. 6a, b). The PreBötC level included sections having the IO-S (Fig. 6c, d), and the VRG level included sections caudal to the IO-S (Fig. 6e, f) (Ruangkittisakul et al. 2014; Stornetta et al. 2006).

### Anatomical identification of CSC regions

**Fig. 7:**
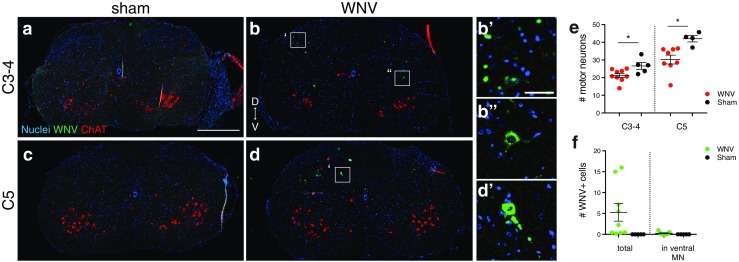
Neuronal survival and WNV IR in the CSC. **a-d** Representative images of CSC at the C3-4 and C5 levels from a sham-infected mouse and the WNV-infected animal having the most WNV IR (animal C1H). Sections were labeled with antibodies against WNV (*green*) and ChAT (*red*, motor neurons). **b’, b”, d’** Close-ups of WNV IR cells in **b** and **d** that include examples of infected cells with neuronal (**b”, d’**) and glial (**b’**) morphology, and the location in the dorsal horns where WNV IR was frequently localized in animal C1H (**b’**). **e **Average number of ventral motor neurons (ChAT+) per section at each level. Results were significantly different between groups for the C3-4 and C5 levels (*, p < 0.05). **f **Average number of WNV IR cells per section for the whole section (total) and in ventral motor neurons. *D, *dorsal; *V,* ventral. *Scale bars* = 500 mm (**a-d** same scale), 50 mm (**b’, b”, d’** same scale). *Error bars* in **e** and **f** represent group mean ± SEM (color figure online)

CSC levels were identified by the location and size of ChAT+ clusters in the ventral spinal cord. At C2, there are small clusters in a ventral-medial location; at C3–C4, there are small clusters in ventral-lateral locations (Fig. 7a, b); and at C5, there are large lateral clusters (Fig. 7c, d) (Watson et al. 2009). Because PMNs are located in C3–C5 (Watson et al. 2009), we focused our analysis on this region. We analyzed the C5 region separately because it contains more neurons than the C3–C4 regions.

### Scoring of histopathologic lesion severity

The severity of histopathologic lesions in the brainstem and CSC of each animal were scored according to the following semiquantitative scale: no lesions (0), minimal (1), mild (2), moderate (3), and severe (4). If the approximate number of lesions per section was <5 and/or <10 % of the area was vacuolated, the score was minimal (1). If the approximate number of lesions per section was 5–10 and/or 10–25 % of the area was vacuolated, the score was mild (2). If the approximate number of lesions per section was 10–20 and/or 25–50 % of the area was vacuolated, the score was moderate (3). If the approximate number of lesions per section was >20 and/or >50 % of the area was vacuolated, the score was severe (4). At least five sections were analyzed for each score. Analysis of the CSC focused on the C3–C4 levels, and analysis of the brainstem focused on the BötC and PreBötC levels.

### Quantification of nonmotor neurons in the RCs

The respiratory column (RC) region was defined as a wedge shape having its apex at the nucleus ambiguus and base at the ventral edge of the brainstem (Fig. 6c). This region is notably larger than the BötC and PreBötC complexes but was chosen to avoid errors arising from trying to sample from too small of an area. Using the cell counter plugin in ImageJ, the number of NeuN+, ChAT− cells (nonmotor neurons) in each wedge was counted. The count from each wedge was assigned an anatomical level as described above. Counts at each level were averaged and multiplied by 2 to get the average total number of nonmotor neurons in RCs per section at the BötC, PreBötC, and VRG levels. Averaged values were multiplied by 2 because there were two RCs per section and each count represented the number of neurons in one RC.

### Quantification of motor neurons in the 12N

The cell counter plugin was used to count the number of ChAT+ cells (motor neurons) in the 12N in each section through the middle of the 12N. Sections through the rostral or caudal end of the nucleus had obviously lower numbers of cells and were thus excluded. Counts from each section were averaged to get the average number of motor neurons per section through the 12N.

### Quantification of motor neurons in the CSC

The cell counter plugin was used to count the number of ChAT+ cells (motor neurons) in the ventral spinal cord. Sections were assigned a level as described above. Counts at each level were averaged to get the average number of motor neurons per section at the C3–C4 and C5 levels.

### Quantification of serotonin (5HT) neurons

**Fig. 8:**
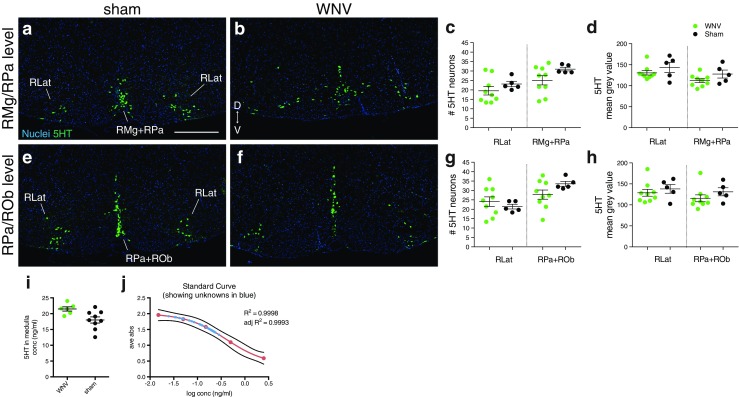
Serotonergic neurons and 5HT levels in medullary raphe nuclei. **a**, **b**, **e**, **f** Representative images of brainstem at the level of the RMg and ROb from WNV- and sham-infected mice labeled with an antibody against serotonin (5HT, *green*). **c**, **g** Average number of 5HT+ neurons per section in each raphe region at each level. **d**, **h** Average mean gray value of 5HT+ pixels per section in each raphe region at each level. **i** Results from a 5HT ELISA showing the amount of 5HT present in the medulla of six WNV- and nine sham-infected animals. **j** Standard curve from 5HT ELISA showing all unknown samples (*blue*) fell within the range of the standard curve. *D* dorsal, *V* ventral, *RLat* lateral raphe, *RMg* raphe magnus, *RPa* raphe pallidus, *ROb* raphe obscurus. *Scale bar* = 500 μm (**a**, **b**, **e**, **f** same scale). *Error bars* in **c**, **d** and **g**–**i** represent group mean ± SEM (color figure online)

The raphe nuclei in the medulla have two distinct distributions depending on the level (Franklin and Paxinos 2007). At rostral levels, there is very little raphe obscurus (ROb) and the raphe magnus (RMg) and raphe pallidus (RPa) occupy a triangle at the ventral midline (Fig. 8a). At caudal levels, the RMg disappears and the ROb is obviously present and forms a line of cells along the midline that is difficult to separate from the RPa (Fig. 8e). The lateral raphe (RLat) are equally apparent at both levels. Three sections representing each level were chosen for quantification. Regions of interest (ROIs) were defined for RLat and RMg+RPa at rostral levels and RLat and ROb+RPa at caudal levels. Using the cell counter plugin, the number of 5-hydroxytryptophan (5HT)+ cells in each ROI was counted. Counts were averaged to get the average number of 5HT neurons per section for each ROI. For mean gray values, images were thresholded and the mean gray value of thresholded pixels within each ROI was obtained.

### Quantification of WNV+ and CD3+ cells

The number of WNV+ or CD3+ cells on each section was counted under the microscope. If the number of cells was numerous, an image was taken so the cell counter plugin could be used to assist with counting, but positive cells were verified under the microscope if they were not obvious on the image. Counts were assigned a level and averaged as described above.

### Quantification of GFAP and iba1

The whole area to be included in quantification, excluding artifacts, was defined by an ROI. For parenchymal GFAP analyses, the parenchymal region excluding the outer rim was analyzed. Single-channel images were thresholded to select pixels with signal, and thresholded pixels within the main ROI were measured to obtain the total area and mean gray value of iba1 or GFAP+ pixels within the ROI. The area of positive pixels was divided by the total ROI area to determine what proportion of the ROI was positive for the antibody. The average proportional area and mean gray value per section at each level was calculated as described above. Averages denoted “all” included sections from all levels in the average calculation.

### Heat map generation

Data for individual animals are shown in heat map style to make it easier to visualize how far from “normal” each parameter was for each animal (Figs. 1, 3, and 5). For each parameter, the mean and standard deviation (SD) of the sham values were calculated. Then, the value for each animal (including the sham-infected animals) was expressed in terms of standard deviations of the sham mean by the following formula: (animal value − sham mean) / sham SD. Then, the raw values were shaded in the heat map as follows. If the value was within 1 SD of the sham mean, the value was shaded green. If the value was over 1 SD of the sham mean, the value was shaded blue, and if the value was under 1 SD of the sham mean, the value was shaded red. The darkness of the red or blue indicates how much the value exceeded or fell below 1 SD of the sham mean.

### 5HT ELISA analysis of the medulla

A 5HT ELISA kit, with a sensitivity of 0.005 ng/mL, was used to measure serotonin (Rocky Mountain Diagnostics, Inc., BA E-5900). Nine animals were infected with WNV and nine with sham inoculum as described above. Body weight analyses indicated a productive infection, with three WNV-infected animals dying by 8 DPI. The six remaining WNV- and nine sham-infected animals were sacrificed by CO_2_ asphyxiation for analysis on 8 DPI. To isolate the medulla, the brains were removed from the skull and kept moist with PBS in a Sylgard dish. The cerebrum, the cerebellum, residual spinal cord, and the pons were removed with a scalpel. The remaining medulla was transferred to 1 mL of PBS containing 0.01 % Triton X-100 and 1 % of the 10 % stabilizer solution supplied with the ELISA kit, as recommended by the manufacturer. The tissue was homogenized, kept on ice until all samples were collected, then stored at −80 °C. Before the test, all samples were thawed to RT, spun (10,000 rpm, 5 min, 4 °C) to pellet debris, and supernatants were transferred to fresh tubes. Supernatants were diluted 1/100 with the diluent supplied in the kit, and 100 μL of the diluted sample was used for the ELISA, which was then completed according to the manufacturer’s instructions.

### Statistics

**Fig. 9:**
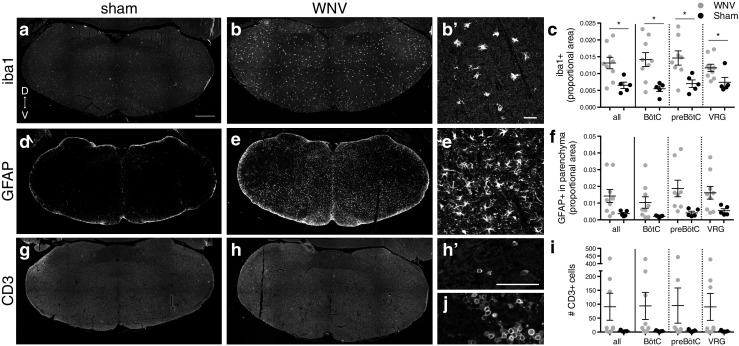
Host response to virus in brainstem. **a, b, d, e, g, h** Representative images of brainstem from WNV- and sham- infected mice labeled with antibodies against iba1 (**a, b,** microglia/activated macrophages), GFAP (**f, e,** astrocytes), and CD3 (**g, h,** T-cells). **b’, e’, h** Close-ups of labeled cells in **b, e,** and **h** showing cell morphology of activated microglia (**b’**), reactive parenchymal astrocytes (**e’**), and T-cells (**h’**). **j** Spleen (positive control tissue) labeled with antibodies against CD3. **c, f, i** Average proportion of the brainstem having iba1+ pixels (**c**), proportion of the brainstem parenchyma having GFAP+ pixels (**f**), and number of CD3+ cells (**i**) per section. *All,* includes sections from all levels in average; *BötC, preBötC,* and *VRG,* include only sections at each level in average. *D, *dorsal; *V,* ventral; *, p < 0.05. Scale bars = 500 mm (**a, b, d, e, g, h** same scale), 50 mm (**b’, e’** same scale, **h’, j** same scale). *Error bars* in **c, f** and** i** represent group mean ± SEM

Data were analyzed in Prism (GraphPad Software, Inc.) for statistical significance using two-way ANOVAs with post hoc *t* tests or *t* tests. Tests comparing WNV vs sham groups were performed for all data shown in graphs except for regional GFAP and CD3 data shown in Fig. 9 f, i. The groups were not significantly different unless noted.

### Hamster methods

Nerve recording surgeries in the hamster were performed similar to the mouse surgeries, with the following modifications. Anesthesia with urethane was initially supplemented with 0.8–1 % isoflurane so that surgery could begin sooner. Isoflurane levels were adjusted to maintain stable HR and BP. After 30–45 min on urethane, isoflurane and was gradually turned off in a manner that ensured stable HR and BP and a lack of withdrawal to paw pinch. The external jugular vein and common carotid artery were cannulated before tracheotomy and ventilation. Animals were ventilated (Harvard apparatus ventilator Model-683) with 100 % O_2_ (1.5 L/min) at a tidal volume of 0.7 mL/100 g and rate of 70–80 bpm. Neuromuscular blockade was achieved with pancuronium bromide (1 mg/kg i.v. initial dose, supplemented with 0.5 mg/kg every hour). After the PN and 12N were isolated and placed on the electrodes, hamsters were hyperventilated at 110 bpm until PN activity disappeared and were allowed to stabilize in “phrenic apnea” for 30 min. The ventilator rate was then turned down 5 bpm every 5 min until 40 bpm was reached to challenge the animals with increasing CO_2_ levels. This was done to assess the full range of responses from apnea to baseline to high CO_2_ levels. Finally, animals were challenged with 5 % CO_2_ (21 % O_2_ with N_2_ balance) at 40 bpm. Tissues were prepared for histological analysis similar to mouse tissues, except that the hamster brain matrix (ASI, hamster RBM-9000C) was used to cut the brain into sections. Sections were stained with H&E and antibodies against WNV, NeuN, ChAT, and 5HT (Table 1) similar to the way mouse sections were stained. Quantification was done using the cell counter in FIJI (Schneider et al. 2012).

## Results

### Nerve recordings, amplitude parameters

We analyzed PN and 12N activities in WNV- versus sham-infected mice at 8 and 10 DPI, time points at which many mice develop serious illness. Four experiments, A, C, D, and E, were conducted (Fig. 1). Survival and weight loss data demonstrated that disease was very apparent in WNV-infected mice. One of 21 mice was dead on 8 DPI in the 8 DPI cohort, and 18 of the remaining 20 mice had percent weight loss values below 2 SD of the sham mean (Fig. 1). Six of 12 mice were dead on 10 DPI in the 10 DPI cohort, and all of the remaining six mice had weight loss values below 3 SD of the sham mean (Fig. 1). To promote rigor and transparency in scientific reporting, Fig. 1 shows which of the surviving mice were included in the PN activity, 12N activity, and histological datasets, why certain animals were excluded, and how representative the datasets are of the whole set of infected mice.

The PN amplitude between WNV- and sham-infected animals was clearly different (Fig. 2a, b). At baseline, PN amplitude was not significantly different between the groups, but after CO_2_ exposure, PN amplitude was clearly blunted in the WNV-infected animals compared with sham (*p* ≤ 0.005; Fig. 2b). These results are exemplified in representative nerve traces from a sham-infected mouse (animal C2C) and a WNV-infected mouse (animal C1H) (Fig. 2a). Values for each individual animal are shown in a heat-map style in Fig. 3. Similar results were obtained for 12N amplitude. There was no significant difference at baseline, but after CO_2_ challenge, 12N amplitude was significantly reduced in the WNV- compared to sham-infected group (*p* ≤ 0.01; Fig. 2c). Two-way ANOVA of the main effect of WNV infection on the amplitude parameters, which considers values at baseline and after CO_2_ challenge together, found that WNV infection has a statistically significant effect on PN amplitude (*p* ≤ 0.01; Fig. 2b), but not 12N amplitude. Thus, PN amplitude was, overall, more affected than 12N amplitude.

When CO_2_ responses were expressed as a percent of baseline, there was no significant difference between the groups for these amplitude parameters (Fig. 2g, h). This suggests that the relative ability of WNV-infected animals to respond to CO_2_ is normal in most animals. Thus, the reduced PN and 12N amplitude after a CO_2_ challenge was likely due to a deficit in pre-motor or motor neuron output rather than a deficit in brainstem chemosensory responses.

### Nerve recordings, time-dependent parameters

There were minimal differences in the PN time-dependent parameters Ti, Te, or burst frequency between WNV- and sham-infected mice (Fig. 2d–f). Two-way ANOVA found no main effect of WNV infection on any of these parameters. Post hoc *t* tests found no statistically significant difference between the groups at baseline or after CO_2_ challenge for any time-dependent parameter, except for baseline Ti (*p* ≤ 0.01; Fig. 2e) which was statistically higher in WNV-infected animals compared to sham. In the context of the rest of the data, however, this may not be biologically relevant.

After exposure to CO_2_, frequency and Ti decreased, while Te increased for both groups. When values after CO_2_ challenge were expressed as a percentage of baseline, there was no statistically significant difference between groups for any of these time-dependent parameters (Fig. 2i–k). These data suggest that WNV infection does not typically affect the breathing rhythm-generating areas of the brainstem, namely the BötC and PreBötC. These data also suggest that brainstem CO_2_ responses are usually normal.

### Histopathology

To determine if PN and 12N amplitude deficits were associated with the presence of histopathologic lesions, we analyzed cross sections of the medulla and CSC from a representative selection of animals (Fig. 1). Histopathologic analysis revealed only mild lesions, although lesions were more severe at 10 DPI than at 8 DPI (Figs. 4 and 5). Nevertheless, we do know that disease was present at the time-points analyzed, because there was substantial weight loss and occasional mortality in each cohort (Fig. 1).

Lesions observed included gliosis/mononuclear and neutrophilic inflammation (Fig. 4g, i, j), which was indicated by an increased number of cells with small nuclei, neuronophagia (Fig. 4h, k, l), neuronal necrosis (Fig. 4m, n), swollen axons (Fig. 4h, i), and some microhemorrhaging (Fig. 4n). Some of the cells in the regions of gliosis/inflammation and neuronophagia had multilobed nuclei characteristic of neutrophils (arrow in Fig. 4l). Immunohistochemistry was done on adjacent sections to further determine the identity of these cells (see “Host response to virus”). Vacuolation in the white and gray matter of the CSC (Fig. 4e) and in midline structures of the medulla was also noted (Fig. 4b). In the CSC, lesions were more concentrated in the ventral horns where motor neurons reside (Fig. 4h, i), but could also be found in all parts, including the dorsal horns (Fig. 4g). In the brainstem, lesions were widely distributed and perhaps more concentrated around the midline at the level of the IO-S (Fig. 4b).

The following scale was used to grade the severity of lesions seen for each animal in each location (CSC or brainstem): none (0), minimal (1), mild (2), moderate (3), and severe (4). None of the tissues were given a score of 4 because the severity of lesions did not meet the criteria for that score (see “Materials and methods”). Most animals scored a 0 for both brainstem and CSC (Fig. 5). Animal D1D, which had a mild PN deficit, had minimal lesions in the brainstem and CSC (score of 1). Animal D1G, which had no PN deficit, had minimal lesions in the brainstem (score 1) and mild to moderate amounts in the CSC (score 2.5). Thus, the location and severity of the lesions did not correlate tightly with PN amplitude results. Animals E2B (score 3 in brainstem and 2.5 in CSC) and E4D (score 2 in brainstem and 1 in CSC) were 10 DPI-animals that died during surgery.

Overall, these data suggest that WNV infection causes neuronal damage and loss, axonal damage, and an inflammatory response. Histopathologic lesions are minimal at 8 DPI, increase at 10 DPI.

### WNV immunoreactivity

Very little WNV IR was found in the brainstem (Figs. 5 and 6h, i, j) or CSC (Figs. 5 and 7). Aside from animal C1H (8 DPI), which had readily detectable WNV IR cells, all the other animals had only sparse numbers. There was slightly more WNV IR in the CSC than in the brainstem and slightly more at 8 DPI than at 10 DPI (Fig. 5). WNV IR cells could be found in any region, but in animal C1H, there was some localization to the dorsal horns in the CSC (Fig. 7b') and the spinal trigeminal nucleus in the brainstem (Fig. 6i, l). Some WNV IR cells appeared to be neurons (Figs. 6k and 7b", d') and some appeared to be glia (Figs. 6l, m and 7b').

### Neuronal survival, respiratory column neurons

To estimate the number of surviving BötC, PreBötC, and VRG neurons, we counted the number of nonmotor neurons in the region of the RCs (Fig. 6a–f). There was a significant decrease in the number of NeuN+, ChAT− cells (nonmotor neurons) in the BötC and PreBötC (Fig. 6a–d, g). The fact that the timing parameters of breathing were mostly normal suggests that the respiratory system is able to compensate for this loss. There was no significant difference in the number of neurons in the VRG region, suggesting that pre-motor neurons are less affected (Fig. 6e–g).

### Neuronal survival, hypoglossal motor neurons

To determine the effect of WNV infection on hypoglossal motor neurons, ChAT+ cells were counted in the 12N and averaged (Fig. 6e–g). No statistical difference in the number of motor neurons was observed between WNV- and sham-infected mice. Thus, any deficits in 12N amplitude do not seem to be associated with motor neuron death. While the animal with the lowest 12N amplitude also had the lowest number of motor neurons in the 12N (animal C1H), motor neuron numbers for the other animals were not correlated with 12N amplitudes (Fig. 5).

### Neuronal survival, phrenic motor neurons

The parameter that best correlated with PN amplitude was the number of ventral motor neurons in the C3–C5 CSC, which contains the PMNs. There was a statistically significant decrease in the number of motor neurons in the C3–C4 and C5 regions (Fig. 7a-e). These results were loosely associated with PN amplitude results. Three of the five animals with a PN deficit had fewer CSC motor neurons than the animal with no PN deficit (Fig. 5). The animals that were the sickest and died in surgery had the lowest CSC motor neuron counts, suggesting that they may have had the lowest PN amplitudes if they had survived long enough to be measured (Fig. 5).

### Neuronal survival, serotonergic neurons

Prior studies have demonstrated that 5HT-expressing neurons in the raphe nucleus modulate respiratory function (Pilowsky et al. 1990a; Verner et al. 2004; Verner et al. 2008) and temperature regulation (Hilaire et al. 2010; Ray et al. 2011). Since WNV-infected mice have respiratory deficits (Morrey et al. 2012; Wang et al. 2013), hypothermia (data not shown), and histopathologic lesions in the midline of the medulla (where the raphe nuclei are located) (Fig. 4b), we quantified the number of 5HT+ cells and the amount of 5HT in the raphe nuclei of the medulla. Although the number of 5HT+ cells and the mean gray value trended downward in several WNV-infected mice (Fig. 8a–h), no statistical significance was found between the groups for any of the nuclei (Fig. 8c, d, g, h). To more rigorously quantify 5HT levels in the medulla, we performed an ultrasensitive 5HT ELISA on medulla tissue. Again, there was no statistically significant difference in 5HT levels between the groups (Fig. 8i, j). These data suggest that WNV-induced respiratory deficits are not due to changes in 5HT concentrations in the caudal raphe nuclei.

### Host response to virus

To determine the identity of the cells identified in the areas of gliosis, inflammation, and neuronophagia in the H&E-stained sections (Fig. 4g–l), sections adjacent to those analyzed for H&E, WNV, and neuronal counts were stained with markers of microglia/activated macrophages, astrocytes, and T cells.

### Host response to virus, microglia/macrophages

**Fig. 10:**
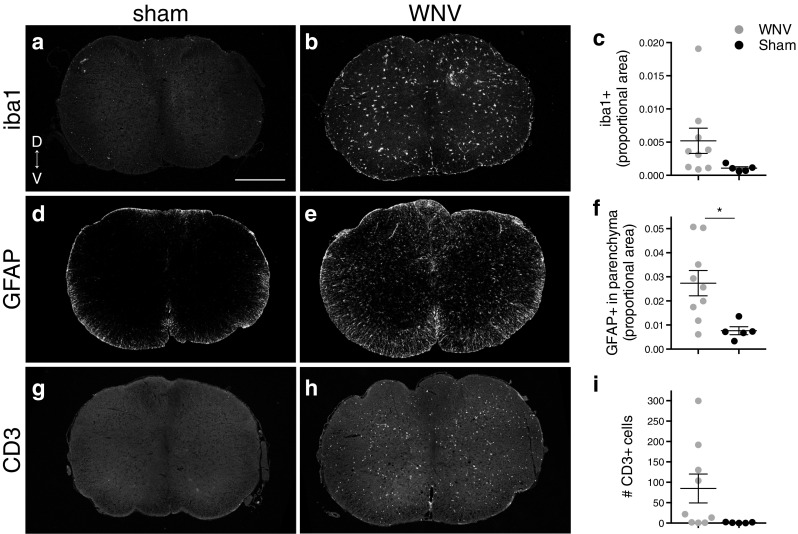
Host response to virus in CSC. **a**, **b**, **d**, **e**, **g**, **h** Representative images of CSC from WNV- and sham-infected mice labeled with antibodies against iba1 (**a**, **b** microglia/activated macrophages), GFAP (**d**, **e** astrocytes), and CD3 (**g**, **h** T cells). **c**, **f**, **i** Average proportion of the CSC having iba1+ pixels (**c**), proportion of the CSC parenchyma having GFAP+ pixels (**f**), and number of CD3+ cells (**i**) per section (where sections from all levels are included in the average, i.e., C3–C4 and C5). Results were significantly different between groups for parenchymal GFAP+ proportional area (*p < 0.05). *D* dorsal, *V* ventral. *Scale bars* = 500 μm (**a**, **b**, **d**, **e**, **g**, **h** same scale). *Error bars* in **c**, **f**, and **i** represent group mean ± SEM

An antibody against iba1 was used to reveal activated microglia, which have higher levels of iba1 than resting microglia (Fig. 9a, b, b'), and macrophages. Many WNV-infected animals had a notable increase in the amount of iba1 IR in both the medulla (Fig. 9a-c) and the CSC (Fig. 10a–c). Iba1 IR was spread evenly throughout the tissue and not localized to a specific region or level (Figs. 9b, c and 10b). The difference between the group averages reached statistical significance for the brainstem (Fig. 9c). Although significance was not reached for the CSC (Fig. 10c), six of nine animals had values that were at least 2 SD above the mean sham value (in fact, all six were over 4 SD above the mean sham value) (Fig. 5).

### Host response to virus, astrocytes

Reactive astrocytes upregulate glial fibrillary acidic protein (GFAP) and develop thicker processes (which are GFAP IR); therefore, a GFAP antibody was used to analyze astrocyte reactivity (Fig. 9d, e, e'). Sham-infected animals had a rim of GFAP IR around the perimeter of both the brainstem and CSC, corresponding to the glia limitans, but very little parenchymal IR (Figs. 9d and 10d). Many WNV-infected animals, however, had a marked increase in the amount of parenchymal GFAP IR in the brainstem (Fig. 9d-f) and CSC (Fig. 10d–f). As with iba1, there was no localization to a specific region or level (Figs. 9e, f and 10e). The difference between group averages reached statistical significance for the CSC (Fig. 10f), but not the brainstem (Fig. 9f). The amount of parenchymal GFAP IR in the brainstem, however, was at least 2 SD above the mean sham amount for six of nine WNV-infected animals (and all six exceeded the mean sham amount by 3 SD) (Fig. 5).

### Host response to virus, T cells

T cell infiltration was analyzed with an antibody to CD3 (Fig. 9h', j). Many WNV-infected animals had an increased number of CD3+ cells in both brainstem (Fig. 9g-i) and CSC (Fig. 10g–i). As with microglia/macrophage activation and parenchymal astrocyte reactivity, T cells were evenly distributed and not localized to a specific region or level (Figs. 9h, i and 10h). Though the differences in group averages did not reach statistical significance because variation in the WNV group was so large (Figs. 9i and 10i), six of nine WNV-infected animals had brainstem values at least 2 SD above the mean sham value (all six were also over 3 SD above the mean sham value), and another six of nine WNV+ animals had CSC values at least 2 SD above the mean (all six were over 5 SD above the mean sham value) (Fig. 5).

Overall, WNV-infected animals tended to have diffuse microglia/macrophage activation, T cell infiltration, and astrocyte reactivity that were not localized to a particular region or rostral-caudal level. There was slightly more of an immune response present in the CSC than brainstem (Fig. 5). Among WNV-infected animals, however, these host-response parameters were not well correlated with PN amplitude deficits (Fig. 5). For example, all animals with PN deficits had high levels of host response except D1A, and of the two animals without PN deficits, only E2D had lower levels of immune host response.

### Hamster data

**Fig. 11:**
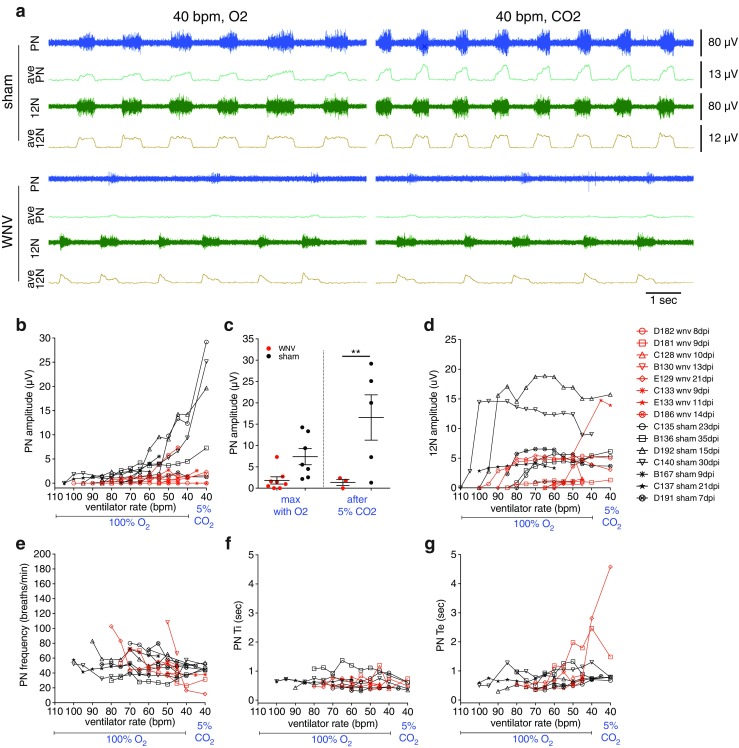
Hamster nerve recordings. **a** Representative PN and 12N recording traces from a WNV- and sham-infected hamster ventilated at 40 bpm, which is itself a “CO_2_ challenge” rate, with 100 % O_2_ versus 5 % CO_2_. Raw traces (80 μV scale) are *blue* (PN) and *green* (12 N), and averaged traces are *teal* (PN, 13 μV scale) and *beige* (12N, 12 μV scale). *Each trace* represents 10 s. **b**, **d**–**g** Average PN amplitude (**b**), burst frequency (**e**), Ti (**f**), Te (**g**), and 12N amplitude (**d**) for 30 s of data at each ventilator rate value and gas type. Each animal is represented separately. WNV-infected animals are shown in *red*, with sham-infected in *black*. **c** Maximal PN amplitude on 100 % O_2_ versus that after 5 % CO_2_ challenge. *Error bars* represent group mean ± SEM. Group means are statistically different after 5 % CO_2_ challenge (**p ≤ 0.01, *t* test) (color figure online)

To ensure that our findings are not unique to mice, we performed similar analyses in hamsters. PN and 12N recordings in hamsters also show that PN, and to a lesser degree, 12N amplitudes appear to be decreased in WNV-infected hamsters compared to sham (Fig. 11a–d). These experiments challenged animals with increasing levels of CO_2_ by decreasing the ventilator rate stepwise from a rate which induces apnea (110 bpm) to a rate which induces a near maximal response (40 bpm), all while animals were being ventilated with 100 % O_2_. This was done to assess the full range of responses from apnea to baseline to high CO_2_ levels. After animals were recorded at 40 bpm with 100 % O_2_, they were challenged with 5 % CO_2_, also at 40 bpm, to check if a maximal response was achieved. Comparison of the maximal PN amplitude on 100 % O_2_ and the amplitude after 5 % CO_2_ challenge shows many WNV-infected animals had values well below the sham mean (Fig. 11c). Means were statistically different for PN amplitude after 5 % CO_2_ (*p* ≤ 0.01).

**Fig. 12:**
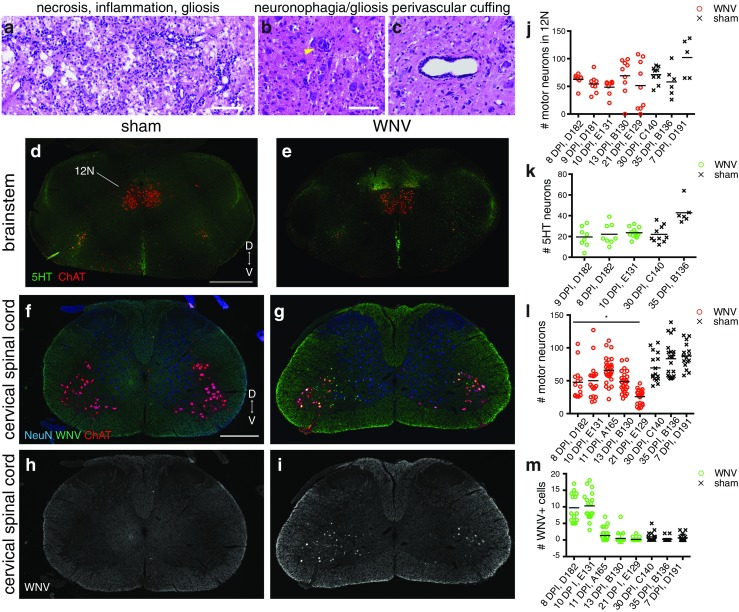
Hamster immunohistology. **a**–**c** Examples of histopathologic lesions in the medulla of WNV-infected hamsters. **a** Necrosis, mononuclear and neutrophilic inflammation, and gliosis. **b** Neuronophagia (*yellow arrowhead*) and gliosis. **c** Mononuclear cell perivascular cuffing. **d**, **e** Representative images of brainstem from WNV- and sham-infected hamsters labeled with antibodies against 5HT (*green*) and ChAT (*red*). **f**, **g** Representative images of CSC from WNV- and sham-infected hamsters labeled with antibodies against NeuN (*blue*), WNV (*green*), and ChAT (*red*). **h**, **i** WNV channels from **f** and **g** shown separately in *white*. **j**–**m** Quantification of the number of motor neurons in the 12N (**j**), 5HT+ neurons in the medulla (**k**), motor neurons in the CSC (**l**), and WNV+ cells in the CSC (**m**). *Dots* represent the count for each section analyzed, and *bars* represent the mean count/section for each animal. The mean of WNV- versus sham-infected means was statistically different for number of motor neurons in CSC (*p ≤ 0.05, *t* test). *D* dorsal, *V* ventral. *Scale bars* = 100 μm (**a**–**c** same scale), 1000 μm (**d**, **e** same scale), 500 μm (**f**–**i** same scale) (color figure online)

Histopathologic lesions and the amount of WNV IR in hamster brainstems and CSCs was typically more severe than what was seen in mouse. Histopathologic lesions included widespread gliosis/mononuclear and neutrophilic inflammation (Fig. 12a), neuronophagia (Fig. 12b), and inflammatory cell perivascular cuffing (Fig. 12c). WNV IR was high at 8–10 DPI and dropped by 11 DPI (Fig. 12h, i, m). As with the mouse, there was not a significant decrease in the number of 12N motor neurons or 5HT+ neurons in the medulla (Fig. 12d, e, j, k). As with the mouse, there was a significant decrease in the number of motor neurons in the CSC (Fig. 12f, g, l), consistent with the observed decrease in hamster PN amplitude. Additionally, the number of CSC motor neurons may decrease over time (from 11 DPI to 21 DPI) (Fig. 12l).

These data suggest that the effects of WNV on the neurological control of respiration is not unique to mice, but may be generalized to all susceptible rodents and perhaps to humans.

## Discussion

The main findings of the current study are as follows. First, there was a clear blunting of PN amplitude in WNV-infected animals. Second, immunohistologic analysis showed that the number of motor neurons in the CSC was significantly decreased in WNV-infected animals compared to shams. Third, histopathologic lesions were localized to the ventral horns of the CSC. Fourth, there was clear evidence of spinal cord infection as microglia/macrophage activation, T cell and neutrophil infiltration, and astrocyte reactivity were all increased after WNV infection (Fig. 10c, f, i). These electrophysiological and immunohistochemical findings confirm and extend results from our previous diaphragmatic EMG studies (Morrey et al. 2010; Wang et al. 2013), and together support the conclusion that WNV-infection causes deficits in the function of lower motor neurons of the respiratory system.

We did not find a clear pattern of cell death, virus burden, or inflammatory responses that could explain why certain animals had more or less severe PN deficits (see Fig. 5). For example, animal C1F, which had the lowest PN amplitude, had minimal cell death and histopathologic lesions in the CSC, but had levels of microglia, astrocyte, and T cell responses over 3 SD above normal. However, animal D1G, which had a normal PN amplitude, had a similar profile, but also had more WNV and more severe histopathologic lesions in the CSC. Animal D1A, which had the second lowest PN amplitude, had a radically different profile. Microglia, astrocyte, and T cell responses were normal (all within 1 SD of the sham means), but it had higher levels of cell death in the CSC (over 1 SD of the sham mean at C3–C4 and over 3 SD of the sham mean at C5). This could mean that multiple factors impact PN function. WNV infection of and replication in motor neruons could directly affect their function both before and certainly after causing cell death, and the inflammatory responses to the virus could indirectly impact motor neuron function with and without causing cell death both during and after virus clearance. Another possibility, however, is that WNV is impacting PN function, directly or indirectly, by causing myelin and/or axonal degeneration, dendritic pruning, or neuromuscular junction dysfunction. The absence of clear immunohistological correlates of PN deficits then indicates a need for further investigation into other parameters that might be more suitable indicators of PMN dysfunction, such as markers of neuronal damage, PN axonal degeneration, de-myelination, and motor end-plate abnormalities.

We did not identify dramatic deficits with brainstem respiratory centers governing breathing rhythm/pattern generation or responses to changing CO_2_ levels. Overall, 12N amplitude was not significantly different from sham. Two-way ANOVA found no main effect of WNV infection on 12N amplitude, though post hoc *t* tests showed it was significantly reduced after CO_2_ challenge, possibly due to motor neuron or axonal dysfunction. Responses to CO_2_, when expressed as a percent of baseline, were normal, indicating that brainstem chemoreceptor responses were intact. There were no apparent differences in the timing parameters, suggesting that BötC and PreBötC functions were typically intact. These results are supported by immunohistologic analyses that found no significant neuronal loss in the 12N or VRG, which contain motor and pre-motor neurons, respectively. Though our analyses found no significant deficits in PreBötC or BötC function between WNV- and sham-infected groups, there were fewer neurons in these areas, suggesting that there could be damage that was not severe enough to evoke a strong deficit at the time points analyzed. This may, however, have played a role in producing a deficit in the few WNV-infected animals that had abnormal values for the timing-parameters (Figs. 3 and 5). There were mild histopathologic lesions localized to midline structures of the medulla, which contain raphe nuclei that modulate respiration, but no decrease in the amount of 5HT or number of serotonergic neurons was found in the medulla. As with the CSC, microglia/macrophage activation, astrocyte reactivity, and T cell and neutrophil infiltration were elevated in the brainstem (Fig. 9 (C, F, I)), but were not localized to any particular nucleus or region, and were not tightly correlated with neuronal loss or PN amplitude. It is possible that other brainstem functions were affected. Of note, however, the responses of blood pressure and heart rate to CO_2_ challenge were normal in this study (data not shown).

Taken together, the data suggest that brainstem dysfunction is not a major contributing factor to WNV-induced respiratory deficits. Our previous results with diaphragmatic EMG responses (Wang et al. 2013) suggested that there may have been brainstem dysfunction, but this approach was not sensitive enough to allow us to observe an EMG signal at baseline or after CO_2_ challenge in some WNV-infected animals. This led us to hypothesize that WNV-infected animals are unable to respond to changes in CO_2_. This hypothesis was specifically tested in this study. Based on our results here, the lack of EMG signal in the prior study (Wang et al. 2013) appears to have been due to the low sensitivity of the diaphragmatic EMG recording, and perhaps due to the depressive effects of isoflurane on the respiratory system (Flecknell 2009). By recording directly from the PN in the current study, we were able to detect PN activity at both baseline and after a CO_2_ challenge. This allowed us to express results after CO_2_ exposure in terms of percent of baseline values. In doing this, we found that while PN amplitudes were reduced in WNV-infected animals, their ability to respond to CO_2_ was intact.

Immunohistologic analyses revealed very mild WNV IR or histopathologic lesions in the brainstem or CSC of WNV-infected mice at the time points analyzed (8 and 10 DPI). One possible explanation is that most WNV IR occurred before 8 DPI and more severe histopathologic lesions developed after 10 DPI. Thus, it may be beneficial to analyze more time points. Another explanation is that WNV expression was below the limit of detection; therefore, a more sensitive assay, such as in situ hybridization, may be of benefit to localize WNV infection. In support of this, a previous study using RT-PCR detected a wave of WNV RNA in brainstem tissues at 6–9 DPI (Hunsperger and Roehrig 2006). Another approach would be to perform a more careful analysis of WNV localization in hamsters, since they exhibited more WNV IR and histopathologic lesions. In the one mouse (animal C1H) that did have a substantial amount of WNV IR, there was some localization to the dorsal horns of the CSC and the spinal trigeminal nucleus in the brainstem, which are sensory areas rather than the expected motor areas. This could be an anomaly and partially due to problems with detection, but this localization is supported by the Hunsperger study (2006) which found virus in dorsal root ganglia and the spinal trigeminal nucleus and tracts. Based on this, they hypothesized that WNV infects the central nervous system by traveling up sensory fibers which innervate the skin where mosquito bites occur. Our earlier studies (Samuel et al. 2007; Wang et al. 2009b), however, suggest that WNV travels up motor tracts in the spinal cord and not sensory tracts. Future work in this area is merited.

Taken together, these data substantiate the previously hypothesized idea that there is a deficit in lower motor neuron/PN function following WNV infection and refute the role of brainstem chemosensory responses or breathing rhythm generation in WNV-induced respiratory dysfunction in two rodent species. Future efforts should explore the time course of the disease and include chronic stages, since several neurological sequelae affecting respiration such as Guillian-Barré syndrome and myasthenia gravis take time to develop (Leis and Stokic 2012). We should also delve deeper into the mechanistic causes of peripheral nerve dysfunction and explore markers of neuronal dysfunction, synaptic pruning (Vasek et al. 2016), axonal degeneration, demyelination, and neuromuscular junction abnormalities.
